# Advances in Wearable Stethoscope Technology: Opportunities for the Early Detection and Prevention of Cardiovascular Diseases

**DOI:** 10.7759/cureus.75446

**Published:** 2024-12-10

**Authors:** Kay M Roh, Ayoola Awosika, Richard M Millis

**Affiliations:** 1 Medicine, American University of Antigua, St. John's, ATG; 2 General Practice, University of Illinois, Chicago, USA; 3 Physiology, American University of Antigua, St. John's, ATG

**Keywords:** cardiovascular disease, early detection, medical devices, preventive medicine, self-diagnostic device, stethoscope, wearable stethoscopes

## Abstract

Wearable technology, including devices like Apple and Samsung watches, Fitbits, and smart rings, has become widely popular. However, while these consumer electronics are readily available, they do not yet meet the accuracy and safety standards required for medical devices by the U.S. Food and Drug Administration (FDA). The COVID-19 pandemic has spurred demand for wearable medical devices, particularly those that can support telemedicine and telehealth. Among these, wearable electronic stethoscopes hold significant promise for early detection and prevention of cardiovascular diseases, which remain the leading cause of death globally. This review highlights the potential of wearable electronic stethoscopes to transform cardiovascular health management by enabling early diagnosis and self-monitoring. Additionally, it examines the current challenges and technological advancements needed to overcome them, underscoring the vital role that wearable electronic stethoscopes could play in improving global health outcomes.

## Introduction and background

Cardiovascular disease (CVD) is the leading cause of illness and death worldwide, encompassing conditions such as heart attacks, strokes, arrhythmias, and heart valve disorders [1]. Ischemic heart disease, responsible for 16% of global deaths, stands as the world’s biggest killer. In the U.S. alone, heart disease led to 702,880 deaths in 2024 [1,2]. Since 2000, CVD has seen the most significant increase in fatalities, with deaths from ischemic heart disease rising by over two million, reaching 8.9 million in 2019 [1,2]. Although CVD was once thought to be mainly a disease of men, one in three women will experience some form of CVD in their lifetime [2]. Despite these statistics, the burden of CVD can be lessened. Most cases of CVD are preventable through early detection and a healthier lifestyle [3]. However, early-stage CVD is often difficult to detect, making timely diagnosis critical to avoid missed cases. Detecting CVD at an early stage increases the chance of preventing its progression to more severe conditions such as ischemic heart disease, atrial fibrillation, and heart failure [3,4]. Early detection also helps reduce healthcare costs, as conditions like heart failure impose a significant financial burden on Medicare due to high rates of sudden cardiac events [5]. In the U.S., heart failure affects around five million people, with 825,000 new cases each year, leading to an estimated $33 billion in annual costs. The lifetime risk of developing heart failure is approximately 20% by age 40 [4].

CVD extends beyond cardiac conditions alone. Research indicates a significant overlap between the mechanisms of Alzheimer’s disease and those of cardiovascular and cerebrovascular diseases [6]. Vascular and cerebrovascular pathology is common in Alzheimer’s patients, though not all individuals with vascular issues develop Alzheimer’s [7]. Studies have also found that older adults with CVD, even excluding stroke, face a higher risk of dementia and Alzheimer’s than those without CVD, with the highest risk seen in individuals with peripheral arterial disease. Extensive peripheral atherosclerosis, in particular, is linked to an elevated risk of Alzheimer’s [7]. Another study further suggests that CVD as a comorbid condition increases the likelihood of Alzheimer’s disease [8]. These findings suggest that preventing the progression of CVD, Alzheimer’s disease, as well as other similar comorbid conditions associated with CVD and cognitive decline, is likely to be possible through early detection of CVD.

Evidence is emerging that wearable electronic stethoscope technologies may have the potential to transform cardiovascular care through early detection and diagnosis, offering a noninvasive, portable, and globally accessible solution. Enhancing traditional acoustic stethoscopes with advanced electronic technologies - such as noise filters, sensors, digital sound processing, machine learning, and wireless connectivity - could enable these devices to function as stand-alone diagnostic tools. This review examines the need for further research and development in wearable electronic stethoscope technology to support early detection and prevention of CVD, the world’s leading cause of death.

## Review

History of stethoscopes

**Figure 1:**
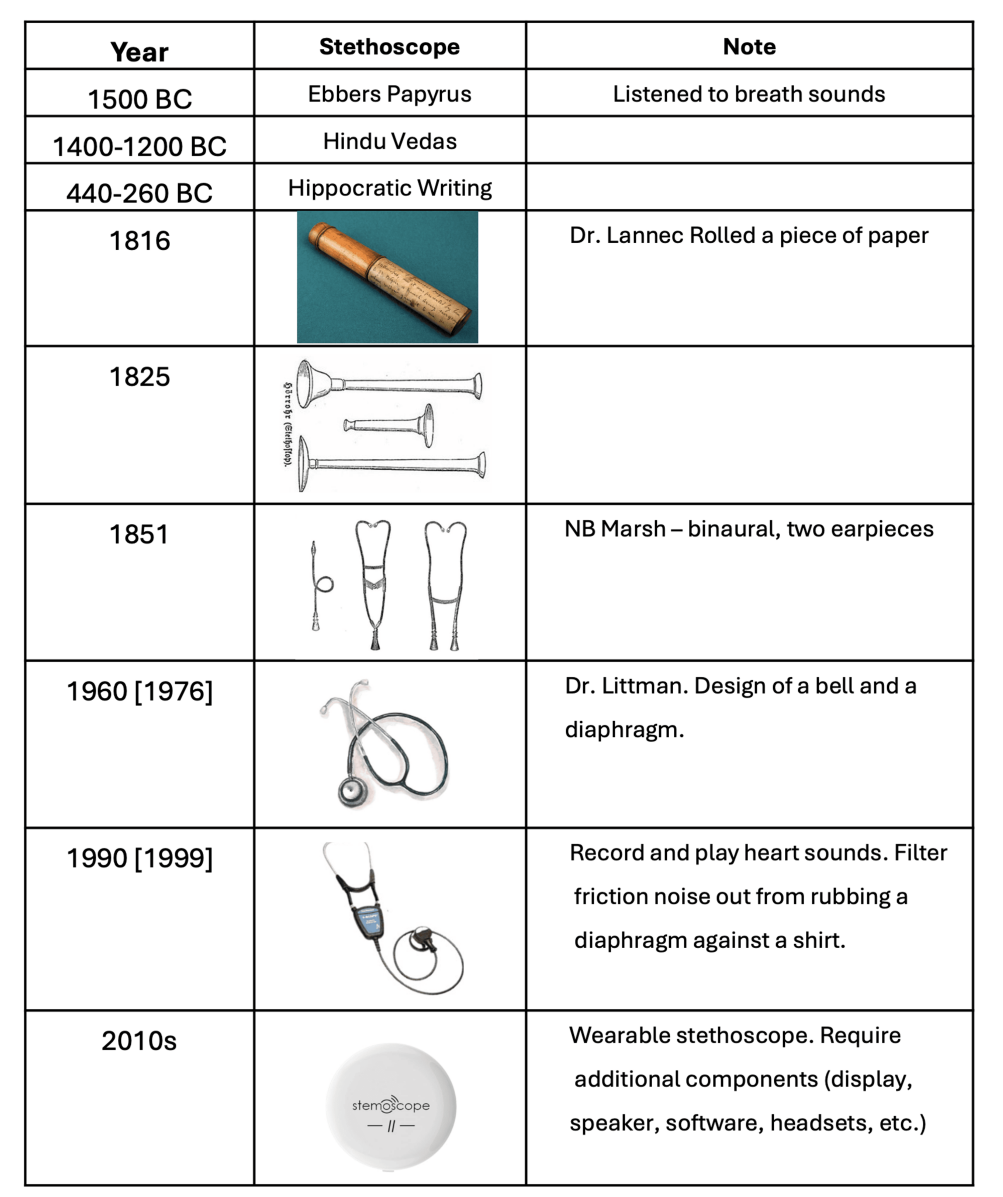
History of stethoscopes Image Credits: Permission has been obtained to reproduce the images.

The traditional acoustic stethoscope is among the most widely used medical devices due to its affordability, simplicity, portability, and low maintenance. The concept of listening to breath sounds dates back to 1500 BCE, as recorded in the Ebers Papyrus, with further mentions in the Hindu Vedas (circa 1400-1200 BCE) and the Hippocratic writings (circa 440-360 BCE) [9]. Figure 1 summarizes the history of the development of the devices known as stethoscopes, which, interestingly, did not appear until the 19th century.

The first stethoscope was invented by Dr. René Laennec, who found it inappropriate to place his head directly on a female patient’s chest to listen to her heart and lung sounds [9,10]. He crafted a simple, rolled paper tube to amplify the sounds, which later proved useful for fetal monitoring in pregnancy [11]. Laennec’s original design was monaural, allowing sound to be heard through a single ear. In 1851, Dr. Arthur Leared introduced the binaural stethoscope, and George Philip Cammann later refined it for commercial use, establishing a design that included a headset, chest piece, and connecting rubber tubing - a design that persists in modern stethoscopes. In 1976, Dr. David Littmann advanced the design with a tunable diaphragm, allowing physicians to switch between high and low frequencies [12,13]. This design, adopted by the 3M Company for the Littmann stethoscope series, incorporated a bell and diaphragm to optimize sound quality.

Acoustic stethoscopes function by transmitting body sounds through air-filled tubes, relying on direct vibrations from the chest piece. Sound energy must be well-preserved to ensure accurate interpretation. In 1999, Dr. Richard Deslauriers, with support from the giant healthcare and medical equipment manufacturer, the Johnson & Johnson Corporation, and in collaboration with a company known for making advancements in audio equipment, the Bose Corporation, developed the Cardionics E-scope. The Cardionics E-Scope could record and playback chest sounds and heartbeats while using insulated tubing to filter external noise [14]. Although innovative, the high cost limited its accessibility for widespread use in clinics and hospitals.

Auscultation and sound frequency

In the field of cardiac auscultation, identifying specific heart sound frequencies is essential for accurate diagnosis and monitoring. Heart sounds encompass a range of frequencies that vary depending on the underlying cardiac event, as shown in Table 1. For effective auscultation, physicians must consider factors like the choice of stethoscope (bell or diaphragm), the specific auscultation site, and patient variables such as age, body composition, and medical history. Experience and training can help practitioners navigate these variables, yet some challenges, such as hearing loss, remain significant [15,16]. Table 1 shows the frequency range of the common heart sounds that are detected by auscultation using standard stethoscopes.

**Table 1:** Heart sounds and characteristics

Sound	Frequency range (Hz)
S1	50 to 500
S2	50 to 500
S3	20 to 200
S4	20 to 200
Murmurs and ejection clicks	Below 300

Hearing loss, particularly among physicians, can impact the ability to detect heart sounds across critical frequency ranges. High-frequency hearing loss can result from noise exposure, aging, and certain medical conditions, while low-frequency loss may be due to age-related changes, structural ear damage, or obstructions within the ear. Studies show that hearing thresholds increase with age, affecting the perception of low-frequency sounds essential for identifying murmurs, clicks, and other cardiac anomalies, as shown in Table 2. Research suggests that approximately 20-38% of physicians over 60 experience varying degrees of hearing loss, with a marked increase in hearing thresholds for frequencies above 2000 Hz in older age groups [17,18]. These findings underscore the need for regular audiology evaluations among older physicians to maintain proficiency in auscultation, as missing low-frequency heart sounds (20-500 Hz) could lead to overlooked cardiac conditions. Table 2 summarizes the common cardiovascular murmur-related conditions associated with the audiometric sound frequencies required for detecting them.

**Table 2:** Heart sounds and frequency ranges

Murmur type	Frequency range
Aortic stenosis	High frequency (>200 Hz)
Mitral regurgitation	Medium to high frequency (200-500 Hz)
Mitral stenosis	Low to medium frequency (50-200 Hz)
Aortic regurgitation	High frequency (diastolic murmur, >200 Hz)
Tricuspid regurgitation	Low to medium frequency (100-200 Hz)
Pulmonary stenosis	High frequency (systolic murmur, >200 Hz)
Ventricular septal defect	Medium to high frequency (200-500 Hz)

Design and evolution of electronic, digital, and wearable stethoscopes

Electronic stethoscopes mimic the design of acoustic stethoscopes (a chest piece with a bell, a diaphragm, or both; a tube; and binaural earpieces). Similar to the current design of acoustic stethoscopes without tubing, binaural earpieces, or both. However, electronics and digital stethoscopes have an additional component to display signals and show the status of stethoscopes. Electronic stethoscopes have revolutionized cardiac auscultation by enhancing the detection and analysis of heart sounds through amplification and digital modification. These devices utilize advanced components, including sensors, digital filters, speakers, and specialized algorithms, which help convert vibrations from the skin into electric signals. The process begins with a sensor placed behind the diaphragm in the stethoscope’s chest piece, where the chest piece translates vibrations into electronic signals. Different transducers are then used to amplify and filter these signals, projecting the refined sounds into headphones or speakers [19-21].

**Figure 2:**
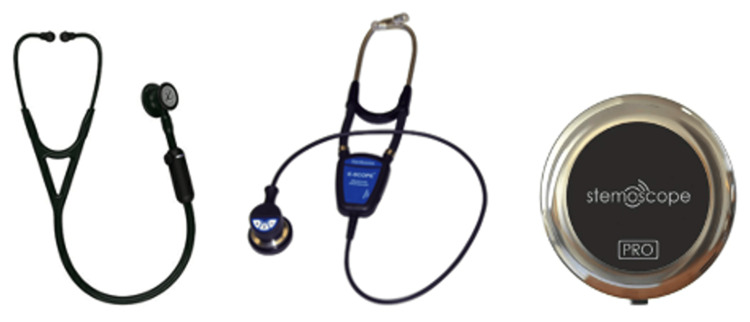
Design of current stethoscopes Left to right: 3M Littmann Core [24], Cardionics E-scope II [26], Stemoscope Pro [27] Image Credits: Permission has been obtained to reproduce the images.

The development of wearable stethoscope technology, which began around 2000-2010, incorporated these electronic advances into a compact, durable form without traditional displays, tubing, or earpieces [22,23]. Designed to be lightweight and comfortable, these disk-shaped devices can be worn for extended periods, from days to a week, conserving battery power due to their streamlined structure. The current, most widely available, and used wearable electronic stethoscopes include 3M Littmann Core [24], Thinklabs One Digital [25], Cardionics E-scope II [26], and Stemoscope Pro [27]. Figure 2 shows the design of the 3M Littman Core, Cardionics E-scope, and Sternoscope Pro.

Wearing positions and methods of wearable electronic stethoscopes

Wearable stethoscopes are evolving to offer continuous cardiac monitoring in a more compact form than traditional devices. Unlike popular consumer wearables like smartwatches and fitness trackers, which provide heart rate estimates but lack medical accuracy, wearable stethoscopes are designed for precise auscultation and are Food and Drug Administration (FDA)-approved for medical use. Typically attached to the chest with medical-grade adhesives, these devices are positioned based on the specific cardiac condition being monitored. For instance, in cases of aortic valve stenosis, a wearable stethoscope might be placed on the upper right sternum, while monitoring for hypertrophic cardiomyopathy could involve devices positioned along the left sternal border, apex, and between. Achieving high-quality sound capture relies on secure and stable contact between the device and the body. Adhesive patches or medical tapes are commonly used, but factors like physical activity, sweat, and skin type can affect device adherence and sound quality. Some wearable stethoscopes also feature flexible chest straps as an additional stabilizing accessory, although these are typically used alongside adhesives to ensure optimal contact between the device’s diaphragm and the skin. This careful placement and secure attachment enhance sound fidelity, helping physicians gather continuous, reliable cardiac data for diagnosis and monitoring.

Current trends in wearable electronic stethoscopes

**Figure 3:**
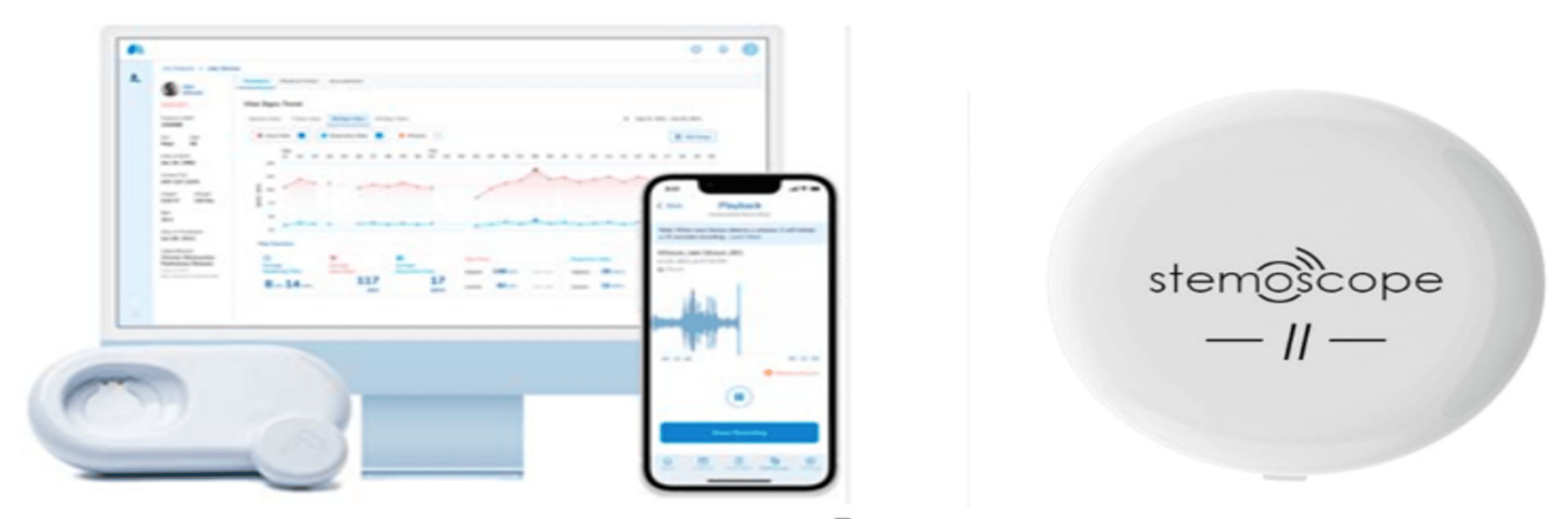
Wearable electronic stethoscope Left to right: AeviceMD [28], Stemoscope II [29]. These wearable devices need displays to communicate effectively with users, making it essential for wearable stethoscopes to integrate with handheld devices or smartphones to display and manage sensor data. Image Credits: Permission has been obtained to reproduce the images.

Wearable stethoscopes are evolving toward more compact and lightweight designs, but they must meet strict standards in accuracy, sensitivity, reliability, security, safety, and liability. Numerous factors influence their effectiveness and usability, including resistance to sweat, impact from physical activity, potential long-term effects, durability, risk of allergic reactions from materials or adhesives, protection against theft, and security of medical data. Additionally, maintaining stable connectivity and ensuring robust data storage are essential for these devices, as they aim to provide continuous, real-time monitoring and long-term data recording. The effectiveness of wearable stethoscopes hinges on high-performance sensors - particularly microphones and accelerometers - ensuring they deliver accurate, reliable results in a practical design. This article reviews the specifications and characteristics of two wearable stethoscopes, the AeviceMD and the Stemoscope II, shown in Figure 3 and Table 3.

**Table 3:** Summary of the digital features of wearable electronic stethoscopes

Electronic stethoscope model	Digital filter setting	Features and design	Electric elements	Algorithms	Price
Aevice MD (Aevice Health, Singapore) [28,30]	20-2,000 Hz frequency range	Tubeless, digital stethoscope that can be either hand-held or attached to the chest using adhesive tape. Diaphragm diameter: 15 mm	Bluetooth	Mobile App and computer software	Not available
Stemoscope II (Hulu device, China) [29,31]	20-2,000 Hz frequency range	Tubeless, digital stethoscope that can be either hand-held or an adhesive tape to be wearable, Diameter: 38 mm, thickness: 12 mm, chrome plated zinc allow the body, non-chill diaphragm, and retention ring, white plastic cover	Digital amplification, bell, diaphragm, and extended range modes, Bluetooth, require Bluetooth abled earpieces, require smartphones for display digital information, own software for mobile apps, store information in cloud service, Internet connection is required	DrStemo App	$100

AeviceMD is a wearable, tubeless device with a compact 15 mm diameter, while Stemoscope II is also wearable but offers fewer features than the Stemoscope Pro and other electronic stethoscopes. Both devices utilize Bluetooth to connect to earpieces and link to cloud services via the Internet. However, specifications for weight and battery life are currently unavailable for both. Stable Bluetooth connectivity is crucial for seamless interaction between wearable stethoscopes, earpieces, and smartphones or handheld devices with mobile applications. Additionally, WiFi must provide fast, secure, and reliable connections to cloud services. Any instability in these connections can disrupt the collection, monitoring, display, storage, recording, security, or sharing of heart sounds with healthcare providers, which may directly affect patient safety. AeviceMD focuses on respiratory and cardiopulmonary monitoring [30]. However, there is a need for further research, clinical studies, reviews, and data specifically related to cardiac auscultation with AeviceMD. According to a U.S. patent, AeviceMD incorporates a patented air conduction sensor and a miniature wearable microphone sensor for cardiopulmonary monitoring [32], though detailed specifications about the sensors or microphones are not publicly available. Stemoscope II is recommended for detecting murmurs within the 200-750 Hz range. Its performance in murmur detection has been compared to echocardiography [33]. A study conducted about Stemoscope II showed a 91% match rate with in-person acoustic stethoscopes in murmur detection. The device also supports tele-auscultation through a self-guided mobile app, which demonstrated comparable results to in-person acoustic stethoscopes with a 90% match rate [34]. In sensitivity comparisons, Stemoscope II tele-auscultation and in-person acoustic auscultation achieved 94% versus 92% sensitivity in higher-detection scenarios and 58% versus 55% sensitivity in lower-detection scenarios [34].

Ang compared a wearable device, AeviceMD, and a wireless stethoscope, 3M Littmann CORE [34], for (1) frequency responses; (2) waveform synchronization; (3) spectral power in different frequency ranges; and (4) clinical relevance. Table 4 shows a high correlation coefficient score between AeviceMD’s and Stemoscope’s frequency response curve from the phantom model’s recording [34].

**Table 4:** Correlation coefficients between frequency response curves and signals from different wearable electronic stethoscope Image Credits: Reproduced from Ang et al. [34]. Open access under Creative Commons License.

	Original sound recorded	Aevice MD	3M Littmann Core	Stemoscope
Original sound recorded by chest auscultation	1.00	0.61	0.73	0.59
Aevice MD	0.61	1.00	0.88	0.91
3M Littmann Core	0.73	0.88	1.00	0.81
Stemoscope	0.59	0.91	0.81	1.00

**Figure 4:**
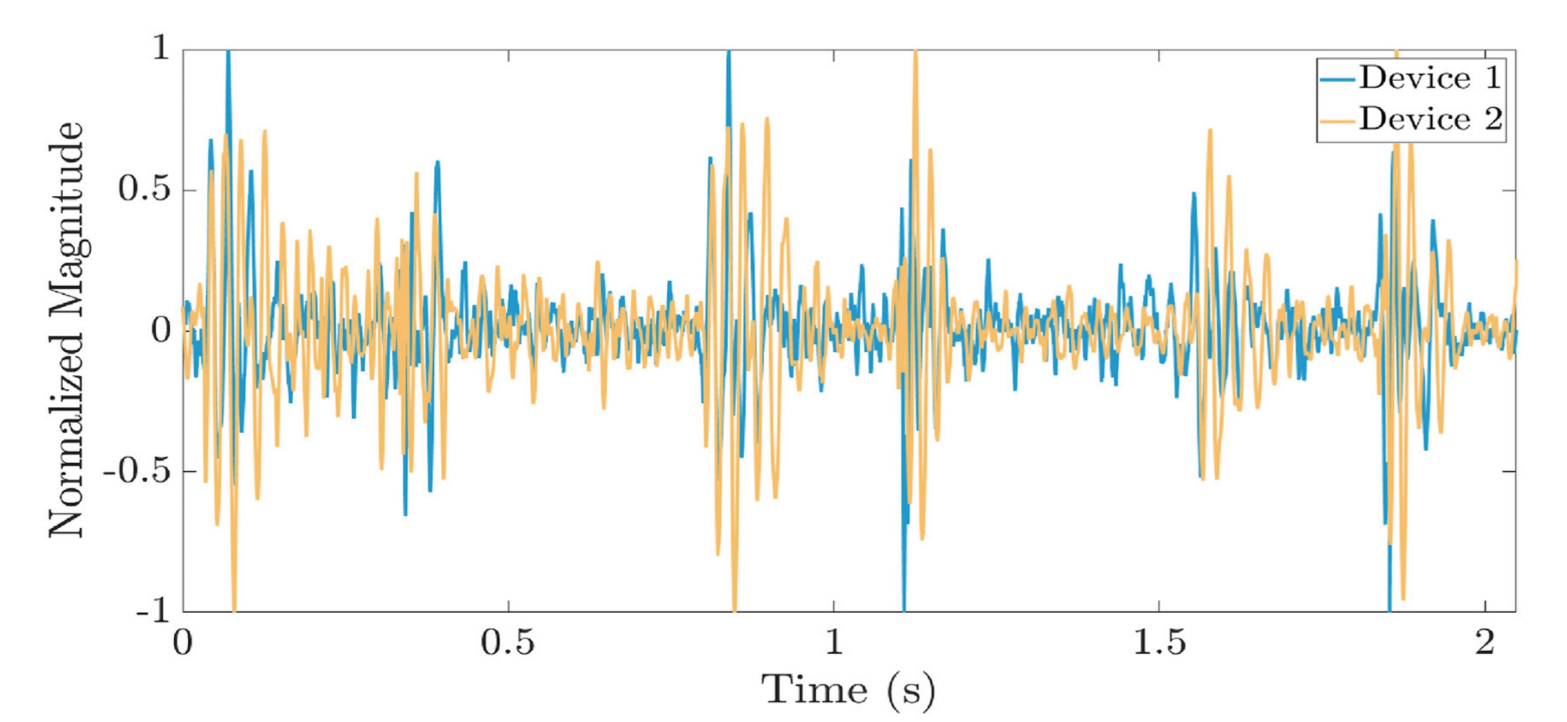
Waveform synchronization between the chest auscultation recordings from AeviceMD and 3M Littmann Model 3200 wearable electronic stethoscopes Image Credits: Reproduced from Ang et al. [34]. Open access under Creative Commons License.

Figure 4 shows a synchronized waveform comparison between the chest auscultation recordings from a wearable stethoscope (AeviceMD) and an electronic stethoscope (3M Littmann CORE) [34].

The synchronization shown in Figure 4 demonstrates that wearable stethoscopes can detect, process, share, and display chest auscultation signals and data with qualities similar to those of other electronic stethoscopes. Table 5 summarizes the results of Ang and associates’ study that recommends devices for specific cardiological clinical relevance and application [34].

**Table 5:** Summary of the devices with the highest and lowest spectral power in different frequency ranges and their respective clinical relevance References: [34-41] Image Credits: Reproduced from Ang et al. [34]. Open access under Creative Commons License.

Frequency	Clinical relevance	Devices with the highest spectral power
<100 Hz	Normal heart sound Stenosis	3M Littmann CORE
100-150 Hz	Normal heart sound	Aevice MD 3M Littmann CORE Stemoscope
200-300 Hz	Heart murmur	Aevice MD Stemoscope
300-400 Hz	Heart murmur	Stemoscope
400-750Hz	Heart murmur	Aevice MD 3M Littmann CORE Stemoscope

The data in Table 5 demonstrate that AeviceMD can be recommended for high-pitched rhonchi, and Stemoscope II can be recommended for detecting heart murmurs because it shows consistent performance in the frequency range of 100-1,000 Hz; 3M Littmann Model 3200 can be recommended for stenosis due to its highest spectral power in the frequency range of less than 100 Hz compared to other devices [34]. Fan et al. compared tele-auscultation and acoustic in-person auscultation with wearable, electronic, and digital stethoscopes [33]. Fan et al. used the Stemoscope as a representative example of an electronic wearable stethoscope, and the result reveals a higher matching percentage (90.6%) than the Eko Core digital stethoscope (87.6%) and JPES-01 (83.3%). However, Stemoscope has only 58% agreement with echocardiography for diastolic murmur (58%). Aortic regurgitation murmurs and mitral stenosis murmurs were correctly heard with Stemoscope II but missed with acoustic in-person auscultation [33]. The results explain and support the motivation of developing electronics, digital, and wearable stethoscopes to detect low-frequency cardiological symptoms, such as heart murmurs, that cannot easily be detected with in-person auscultation. Table 6 summarizes Fan and associates’ analysis of various advanced stethoscopes [33].

**Table 6:** Summary of analysis and results of various modern wearable electronic stethoscope Image Credits: Reproduced from Fan et al. [33]. Open access under Creative Commons License.

Reference	Product name	Tele-auscultation	Smartphone app	Comparison with acoustic stethoscope	Agreement with echocardiography
Fan et al. [33]	Stemoscope	Yes	Yes	90.6%	89% for systolic, 58% for diastolic murmurs
Kalinauskienė et al. [42]	3M Littmann 3200	Not Available	Not Available	Not available	64.17% by cardiologists, 65% by residents
Chowdhury et al. [43]	Eko Core Digital Stethoscope	Yes	Yes	87.6%	Not available
Ghanayim et al. [44]	VoqX	Not mentioned	Not mentioned	Not available	86% for aortic stenosis
Hirosawa et al. [45]	JPES-01	Not Available	Not available	83.3% (vs. stimulator)	Not available
Belmont and Mattioli [46]	ATI	Not Available	Not available	88%	Kappa = 0.55

Benefits and challenges of wearable electronic stethoscopes

Table 7 presents an overview of the benefits and challenges in developing and adopting wearable stethoscopes, highlighting their potential as a valuable addition to the diagnostic tools available to medical practitioners worldwide.

**Table 7:** Benefits and challenges of wearable electronic stethoscopes

Benefits	Challenges
More compact, lightweight	Design is influenced by battery size, electronic components, modules, sensors
Detecting valve regurgitations related to high frequency for older physicians	S3 and S4 (low frequency) for obese patients
Detecting coronary artery disease	Required infrastructures, electricity, high-speed internet, security, and quality of network
Teaching and patient monitoring	Expensive and difficult to replace broken parts
Telemedicine - Reducing medical costs and time for both patients and physicians	The recommendation of using both electronic and acoustic stethoscopes together depends on the patient's conditions
Potential to be convenient, compact, and cheap diagnostic tools for healthcare professionals and physicians	No validation requirements yet
Significant for veterinarians	Less durability than acoustic stethoscopes
Emergency, flight, pandemic	Requires batteries or LED displays
Long-term data for more accurate and better diagnosis and treatment plans	Signal qualities depend on wearing positions and methods

Wearable stethoscopes are designed to perform essential stethoscope functions, including detecting, recording, storing, processing, and outputting high-quality sound data. The effectiveness of these devices largely depends on electronic and digital technologies, which shape their performance, design, and overall quality. Thus, a focus on these technological components is critical. Through sensor technology, wearable stethoscopes can detect and transmit heart sounds, enhancing sensitivity to specific conditions like valve regurgitation, where high-frequency sounds are key indicators [47]. This capability makes wearable stethoscopes valuable for gathering long-term heart sound data for precise diagnosis. Such technology is particularly beneficial for older physicians who may have difficulty detecting high-frequency murmurs due to age-related hearing loss [48-51]. In cases like coronary artery disease, wearable stethoscopes show promise in detecting intracoronary murmurs linked to turbulent blood flow. Studies indicate a sensitivity of 89.5% for diagnosing coronary artery disease affecting over 50% of any significant epicardial artery, with comparable results in animal models, paving the way for veterinary applications [52,53].

However, wearable stethoscopes still face challenges, particularly in detecting low-frequency heart sounds like S3 and S4, which can be easily masked by noise. Traditional acoustic stethoscopes often lose sound quality during transmission through tubes and earpieces, and they lack noise-filtering capabilities, which is problematic in noisy environments or with obese patients, whose thicker body tissues impede sound detection. Wearable stethoscopes mitigate some of these issues by converting sounds directly into electronic data, bypassing the need for tubes and earpieces. Despite this, they still struggle with low-frequency detection. Electronic audiocardiography in cardiovascular assessments of morbidly obese patients, for instance, remains ineffective. Additionally, ensuring firm, stable contact between the stethoscope diaphragm and the skin is crucial for capturing quality sound data, yet this contact can be disrupted by factors such as physical activity, lifestyle, perspiration, wearing method, and device design. For wearable stethoscopes to achieve reliable self-diagnosing capabilities, they must prioritize accurate detection and preservation of original heart sounds across various frequencies and patient conditions. This foundation is essential for advanced diagnostic functions, particularly for applications in challenging environments and with diverse patient populations, including those who are morbidly obese [53].

Digital technology in wearable stethoscopes

Digital technology in wearable stethoscopes enables sound data modification through filtering, amplification, signal processing, storage, and analysis. Clear sound output after filtering is particularly beneficial for physicians and cardiologists working in noisy environments or those with hearing loss. Dr. Tourtier's research found that digital stethoscopes provided better cardiac auscultation quality than traditional acoustic stethoscopes in flight settings [54]. However, signal processing can sometimes alter or lose signals, which may be challenging for practitioners accustomed to the sound profiles of acoustic stethoscopes. Most electronic stethoscopes include output functions on the device head, like the 3M Littmann CORE, which displays heart rate, or the Thinklabs One, which shows various settings. In contrast, wearable stethoscopes lack built-in displays, requiring external devices - such as smartphones, computers, or mobile apps - to access data, which complicates checking for errors or device malfunctions.

Telemedicine and wearable stethoscopes

Telemedicine and telehealth are beneficial for conducting online consultations, monitoring patients for chronic and pediatric patients, travelers, and victims during disasters; providing fast and economical services, especially in emergencies; and improving the quality of education and teaching [54-56]. Wearable stethoscopes have expanded possibilities for telemedicine by allowing extended remote monitoring. This technology is especially valuable for online consultations, managing chronic conditions, monitoring pediatric patients, and providing disaster relief support by offering prompt, affordable care. With battery-powered operations and offline data storage, wearable stethoscopes can continue collecting data even without electricity or internet access, which can be uploaded to the cloud once the connection is restored. This feature is beneficial for immobilized patients, neonates, emergency situations, and individuals with busy lifestyles. However, limited internet infrastructure in some areas restricts data upload and communication with healthcare providers, highlighting the need for expanded connectivity in underserved regions.

Wearable technology and design

Models like the AeviceMD and Stemoscope II are increasingly compact and lightweight, achieved by eliminating direct output functions. Users must use additional devices like smartphones for monitoring and earphones to listen to heart sounds, which can increase costs and discourage long-term health monitoring. The compact design also restricts internal component space, requiring careful selection of storage, battery, and processing components. Higher sampling rates demand greater storage, and advanced data processing necessitates robust electronic support. Battery life is crucial for wearable stethoscopes, as they need enough power for extended use without compromising on compactness. Selecting the right components - battery, memory, and processing units - is essential for ensuring efficient operation and lightweight design. Unlike traditional acoustic stethoscopes, which are simple and inexpensive to repair, wearable stethoscopes are complex and costly to fix or replace, with service policies varying by brand and model.

Discussion

Manufacturers of wearable stethoscopes invest significant effort in replicating the sound characteristics of traditional acoustic stethoscopes while also developing unique algorithms and technologies. Despite capabilities such as amplification, filtering, and algorithmic sound processing, the sound characteristics of wearable stethoscopes differ from those of acoustic stethoscopes, which can create confusion for physicians and healthcare practitioners accustomed to traditional devices. Comparisons between wearable stethoscopes are also challenging, as proprietary software and algorithms are not publicly available, and there are no large-scale validation tools for evaluating their electronic technology [57]. In the U.S., electronic and wearable stethoscopes are classified as Class II devices (special control) by the FDA. For optimal performance, headphones or earphones used with wearable stethoscopes should cover the full range of heart sound frequencies. It is recommended that specific headphone and earphone specifications be provided to healthcare professionals to ensure accurate interpretation; variations in frequency range could lead to data manipulation or omission, which might impact clinical judgment and diagnosis accuracy. The ultimate goal for wearable stethoscopes is to evolve into reliable diagnostic tools capable of interpreting heart sounds and accurately diagnosing various heart conditions, including embryological and valvular disorders and murmurs. However, computer-based heart sound analysis has not yet reached a level of clinical reliability. Achieving this will require extensive clinical studies involving human subjects and real patient examinations [20,58,59]. This article has highlighted the challenges in collecting and preserving high-quality original heart sounds, where machine learning and artificial intelligence could be instrumental in enhancing early detection and self-diagnosis capabilities.

Privacy, security, and protection of patient information

The COVID-19 pandemic accelerated the adoption of modern technologies like telemedicine, cloud data services, wireless and Bluetooth connectivity, mobile applications, and virtual physical examinations in healthcare. These advancements are transforming healthcare delivery, enhancing accuracy, efficiency, user-friendliness, reliability, and adaptability on a global scale [60]. Among these innovations, mobile medical apps and software are leading the way, supporting decision-making, chronic condition monitoring, and improving doctor-patient relationships. These apps integrate various technologies, including artificial intelligence, machine learning, cloud systems, and biosensors, offering significant potential for accurate diagnosis, treatment, and management of diseases while also reducing costs and expanding access to quality care. However, mobile medical apps handle large volumes of sensitive personal information, underscoring the need for strict privacy and security measures. Protecting users’ rights is critical, particularly for apps that collect and share health, medical, and biometric data. To guide developers, the FDA provides a “Mobile Health App Interactive Tool” that outlines relevant federal laws and regulations, emphasizing compliance with the Health Insurance Portability and Accountability Act (HIPAA) and the Children’s Online Privacy Protection Act (COPPA). Notably, HIPAA compliance, updated on July 19, 2023, is foundational, although users consenting to share health information with cloud providers may inadvertently override Fourth Amendment protections [61]. In cases involving cloud storage of health information, courts have applied the “third-party doctrine,” which suggests a reduced expectation of privacy for information shared with third parties. However, recent legal debates are challenging this doctrine’s relevance in the digital age [62-64]. To earn trust, wearable stethoscope manufacturers must prioritize cybersecurity and data protection for patients. Medical record breaches are unfortunately common; according to the U.S. Department of Health and Human Services, 2,867,944 individuals in the U.S. were affected by data breaches in October 2024, with hacking incidents accounting for 88% of these breaches [65]. Building trust with mobile app users, patients, and physicians is, therefore, essential.

## Conclusions

Wearable electronic stethoscopes bridge the data gap between clinics and patients' homes, providing more accurate, long-term diagnostic data. These devices retain the core functions of traditional stethoscopes while leveraging modern technology, making them invaluable tools for telehealth and telemedicine. Although they show great promise as self-diagnostic tools, challenges remain, including patient safety, privacy protection, cybersecurity, potential skin irritation, cost, and internet accessibility. Moreover, wearable stethoscopes are still in the early stages of development. Establishing regulations, validation protocols, and testing methods for wearable stethoscopes is crucial. One study recommends validation settings and tests for electronic stethoscopes to ensure performance across a full range of frequencies and temperatures, which could serve as a foundation for validating wearable stethoscopes under diverse conditions. With further development, wearable stethoscopes could become self-diagnostic tools capable of early detection and prevention of CVDs and related conditions, such as cerebrovascular and neurovascular diseases. While this review focuses on CVD applications, wearable electronic stethoscopes may also have significant potential in diagnosing pulmonary diseases, a topic for future research.
